# Circulating myeloid-derived suppressor cells predict disease activity and treatment response in patients with immune thrombocytopenia

**DOI:** 10.1590/1414-431X20165637

**Published:** 2017-02-16

**Authors:** J. Zhou, Y. Zhou, J. Wen, X. Sun, X. Zhang

**Affiliations:** Hematology Department, The Second Medical College, Shenzhen People’s Hospital, Jinan University, Shenzhen, Guangdong Province, China

**Keywords:** Myeloid-derived suppressor cells, Immune thrombocytopenia, Regulatory T cells, Disease activity, Treatment response

## Abstract

Immune thrombocytopenia (ITP) is a disease characterized by isolated thrombocytopenia. Abnormal effector T cell activation is an important mechanism in the pathogenesis of ITP. Regulatory T cells (Treg) have a strong immunosuppressive function for T cell activation and their importance in the pathophysiology and clinical treatment of ITP has been confirmed. Myeloid-derived suppressor cells (MDSCs) are other immunosuppressive cells, which can also suppress T cell activation by secreting arginase, iNOS and ROS, and are essential for Treg cells’ differentiation and maturation. Therefore, we speculate that MDSCs might also be involved in the immune-dysregulation mechanism of ITP. In this study, we tested MDSCs and Treg cells in peripheral blood samples of twenty-five ITP patients and ten healthy donors. We found that MDSCs and Treg cells decreased simultaneously in active ITP patients. Relapsed ITP patients showed lower MDSCs levels compared with new patients. All patients received immunosuppressive treatment including dexamethasone alone or in combination with intravenous immune globulin. We found that MDSCs’ level after treatment correlated with platelet recovery. Our study is the first that focused on MDSCs’ role in ITP. Based on our results, we concluded that circulating MDSCs could predict disease activity and treatment response in ITP patients. This preliminary conclusion indicates a substantial significance of MDSCs in the pathophysiology and clinical treatment of ITP, which deserves further investigation.

## Introduction

Immune thrombocytopenia (ITP) is an acquired immune-mediated disease characterized by isolated thrombocytopenia, and is characterized by increased platelet destruction that is mediated by autoantibodies. However, abnormal T cell activation has recently become regarded as a more important mechanism in the pathogenesis of ITP (1,2). Regulatory T cells (Treg) are characterized as CD4+/FoxP3+ and suppress abnormal T cell activation by secreting TGF-β, IL-10, and IL-4 (among others), thereby inducing immune tolerance (3). Accumulating data have confirmed the importance of Treg cells in the pathophysiology and clinical treatment of ITP (4–7). As an autoimmune disease, conventional treatments for ITP include high-dose dexamethasone (DXM), intravenous immunoglobulin (IVIg) and other immune-suppressive regimens (8–10). High-dose DXM treatment could expand regulatory T cells in ITP patients, thus suppressing the abnormal T cell activation (11).

Myeloid-derived suppressor cells (MDSCs) are another population of immunosuppressive cells. They are of myeloid origin with immature phenotype and the ability to suppress T cell function. MDSCs expand in response to tumor, infection or inflammation, then circulate and accumulate in the bone marrow, lymphoid tissue, peripheral blood and inflammation sites (12,13). The phenotype of MDSCs is heterogeneous, but these cells are mostly CD33+/CD11b+/HLA-DR- immature myeloid cells in humans (14). MDSCs suppress abnormal T cell activation by secreting arginase, nitric oxide synthase (iNOS) and reactive oxygen species (ROS) (15), thus sharing a similar function with Treg cells. In addition, MDSCs are essential for Treg cell differentiation and maturation (16–19). Because Treg cells are known to be of importance for ITP pathogenesis, we hypothesized that MDSCs might also be involved in the immune dysregulation mechanism of ITP. No study has yet focused on the function and clinical significance of MDSCs in ITP. Therefore, this study aims to preliminarily explore the clinical significance of MDSCs in ITP patients, thereby providing novel evidence of the immune dysregulation mechanism of ITP.

## Material and Methods

### Patients

Peripheral blood samples were collected from 10 healthy donors and 25 patients diagnosed with ITP at the Hematology Department, Shenzhen People’s Hospital. The diagnosis was made according to the international consensus criteria (8). No pregnant donors were enrolled. Written informed consent was obtained from every donor and patient. All experiments were approved by the Ethics Committee of Shenzhen People’s Hospital. As shown in Table 1, of the 25 patients, 14 had a history of thrombocytopenia and relapsed. The other 11 patients were newly diagnosed. Sixteen patients were treated initially with DXM alone at a dose of 10 mg per day, and 9 received IVIg at a dose of 0.4 g·kg^-1^·day^-1^ for 5 days in combination with DXM. Two peripheral blood samples were collected from every patient, separated by Ficoll-Paque method, and prepared for MDSC and Treg detection, one before DXM administration and the other on the sixth day after DXM treatment. The platelet levels were tested before (day 0), 6 days after (day 6), and 11 days after (day 11) DXM treatment to evaluate the treatment response.

**Table:**
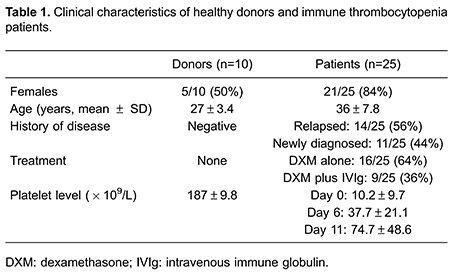

**Figure 1:**
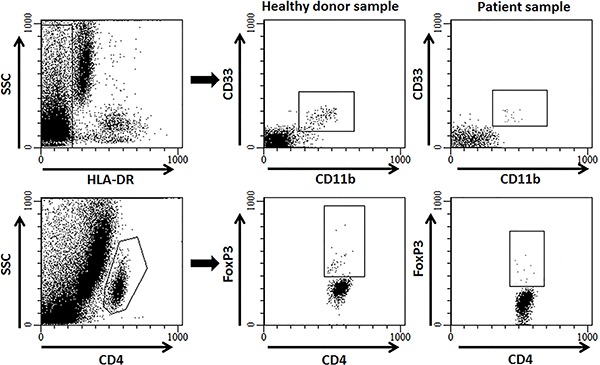
Representative flow cytometry showing labelling and gating strategies for myeloid-derived suppressor cells (MDSC) (*top*) and regulatory T (Treg) cells (*bottom*). MDSCs were defined as CD33+/CD11b+/HLA-DR- cells. Treg cells were defined as CD4+/FoxP3+ cells.

### Flow cytometry

PE-conjugated anti-human CD33, APC-conjugated anti-human CD11b, PerCP-conjugated anti-human HLA-DR and the One-step Staining Human Treg Flow™ Kit were all purchased from BioLegend (USA). The samples were prepared according to the manufacturer’s instructions. The labeled cells were tested on a BD FACSCalibur cytometer and analyzed using FlowJo software version 7.6.1. (Tree Star, USA). In agreement with previous reports, MDSCs were defined as CD11b+/CD33+/HLA-DR- (14), and Treg cells were defined as CD4+/FoxP3+ (20). The labeling and gating strategies are shown in Figure 1.

### Statistical analyses

Statistical analyses were performed using GraphPad Prism (GraphPad Software, USA). Data are reported as means±SD. Student’s *t*-test was used to compare the continuous variables between groups. Linear regression analysis was used to estimate the correlation between variables. Two-sided P values less than 0.05 were considered to be statistically significant.

## Results

### Circulating MDSCs and Treg cells decreased simultaneously in ITP patients

**Figure 2:**
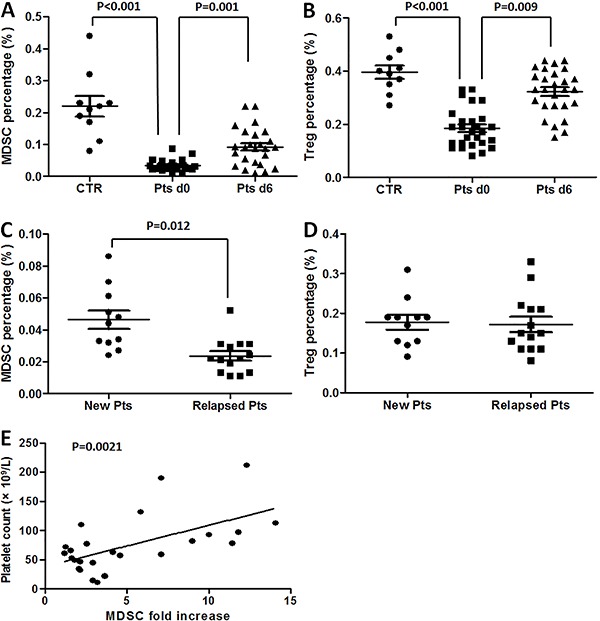
*A*, Blood levels of myeloid-derived suppressor cells (MDSCs), and *B,* regulatory T (Treg) cells were lower in active immune thrombocytopenia (ITP) patients (Pts) on day 0 (d0) compared to healthy controls (CTR). On day 6 (d6) after immunosuppressive treatment, both MDSCs and Treg cells partially recovered. *C* and *D*, Relapsed patients had significantly lower MDSC levels compared to newly diagnosed patients. However, Treg cell levels were similar between the two groups of patients (Student’s *t*-test). *E*, MDSC recovery was defined as the percentage of MDSC on the 6th day after treatment divided by the percentage of MDSC before treatment (or the “MDSC fold increase"). MDSC fold increase and the platelet level on the 11th day after treatment were correlated (linear regression analysis).

In this study, we detected circulating MDSCs and Treg cells simultaneously. We found that both decreased significantly in ITP patients with active disease status, similarly to previous studies (5,11). Representative flow cytometry data from healthy donors and patients are shown in Figure 1. Both MDSCs and Treg cells increased on the 6th day after immunosuppressive treatment (Figure 2A and B). IVIg application did not promote MDSC or Treg cell recovery after treatment (data not shown). This result indicates that MDSCs and Treg cells could be markers for ITP disease activity, while immunosuppressive treatment helps restore the amount of MDSCs and Treg cells in ITP patients.

### Relapsed patients had lower MDSC levels compared to those of the newly diagnosed patients

We compared the circulating MDSCs and Treg cells between newly diagnosed and relapsed patients at admission (day 0). We found that relapsed patients had significantly lower MDSC levels compared to newly diagnosed patients. However, the Treg cell levels were similar between the two groups (Figure 2C and D). This result indicated that MDSC might be a more sensitive marker for disease activity and severity than Treg cells.

### MDSC recovery after treatment could predict the treatment response in ITP patients

Because the MDSCs partially recovered by the 6th day after treatment, we questioned whether the recovery of MDSCs could predict treatment responses. Thus, we defined MDSC recovery as the percentage of MDSC on the 6th day after treatment divided by the percentage of MDSC before treatment (or the “MDSC fold increase”). Through linear regression analysis, we found that MDSC fold increase and the platelet level on the 11th day after treatment were statistically correlated (P=0.0021). In other words, the higher the MDSC increase on the 6th day after treatment, the higher the platelet on the 11th day after treatment (Figure 2E). However, although Treg cells also recovered partially after treatment, the recovery was not significant. Furthermore, we failed to find a similar correlation between Treg cells recovery and platelet count. This result indicates that MDSC recovery after treatment could predict the treatment response. Therefore, we consider the MDSC level to be a prognostic marker in ITP patients.

## Discussion

The role of Treg cells in the pathophysiology of ITP has long been confirmed (4). Many investigators have tested the frequency and function of Treg cells in ITP patients. The majority of studies demonstrated a decreased number of Treg cells in the peripheral blood of ITP patients compared to that in healthy controls (5,6,21). In addition, Treg cell deficiency was also reported as a marker for the disease activity of ITP. For example, a study conducted in Egypt found that acute ITP patients with a severe reduction of Treg cells exhibited prolonged thrombocytopenia, while those with a mild reduction exhibited a brief disease (22). Treg deficiency contributes to the loss of immune tolerance and abnormal activation of T cells in the pathogenesis of ITP.

As another population of immune suppressor cells, MDSCs also play a crucial role in the establishment and maintenance of immune tolerance. As previously reported, MDSCs could suppress immune responses *via* two mechanisms. First, MDSCs could secrete arginase, iNOS and ROS, which directly inhibit T cell proliferation and activation. Thus, MDSCs share similar target cells with and play similar roles as do Treg cells in the immune suppressive microenvironment. Second and more interestingly, MDSCs could induce Treg cell differentiation and maturation *in vivo*. For example, Huang et al. reported that the production of IL-10 and TGF-β by MDSCs was enhanced in response to IFN-γ stimulation, while the accumulation of IL-10 and TGF-β promoted Treg cell differentiation in the tumor microenvironment (23). Serafini et al. (24) reported that this MDSC-mediated Treg cell induction requires arginase but is TGF-β independent. Chou et al. (17 [Bibr B18]) reported that the ability of MDSCs’ to expand Treg cells depends on the B7-H1 molecule. According to these studies, MDSCs are closely related to Treg cells in both function and differentiation. However, the interaction between these two types of cells remains incompletely defined. This study was based on the hypothesis that because Treg deficiency has been confirmed in ITP patients, MDSCs may also be decreased in those patients. In addition, because MDSCs regulate Treg cells in the immunosuppressive network, MDSCs should decrease and recover earlier than Treg cells in ITP patients.

In this study, we found that circulating MDSCs decreased in ITP patients with active disease, similar to Treg cells. We also found that MDSCs decreased more profoundly in relapsed ITP patients than did Treg cells. Additionally, the recovery of MDSCs correlated with platelet recovery after treatment. Our findings support our hypothesis. We believe that our results have important pathophysiological and clinical significance for ITP. First, the decease of MDSCs in ITP patients indicates that MDSCs play an important role in ITP pathogenesis. The pathogenic role of MDSCs in ITP might be the direct inhibition of T cell activation or the indirect activation of Treg cells or other immune-suppressive cells. Second, our finding that MDSCs could act as a prognostic marker for treatment responses may have great value for clinical practice. In future clinical work, we will continue to monitor MDSC levels during treatment. For patients with an unsatisfactory recovery of MDSCs after treatment, whether an early intensification of immunosuppressive treatment would provide a benefit in disease control is an interesting question for further study.

In conclusion, this study is the first focusing on the clinical importance of MDSCs in ITP. Our study showed a reduction in circulating MDSCs in ITP patients that correlated with disease activity and treatment response. This observation primarily demonstrated a substantial significance of MDSCs in the pathophysiology and clinical treatment of ITP, which deserves further investigation.
